# Morphological characteristics of vasculogenic mimicry and its correlation with EphA2 expression in gastric adenocarcinoma

**DOI:** 10.1038/s41598-019-40265-7

**Published:** 2019-03-04

**Authors:** Hee Sung Kim, You Jin Won, Ju Hee Shim, Hyun Ji Kim, Jihun Kim, Hea Nam Hong, Byung Sik Kim

**Affiliations:** 1 Department of Gastric Surgery, Asan Medical Center, University of Ulsan College of Medicine, 88, Olympic-ro 43-gil, Songpa-gu, Seoul, 05505 Republic of Korea; 2 Department of Anatomy, University of Ulsan College of Medicine, 88, Olympic-ro 43-gil, Songpa-gu, Seoul, 05505 Republic of Korea; 3 Department of Pathology, University of Ulsan College of Medicine, Asan Medical Center, 88, Olympic-ro 43-gil, Songpa-gu, Seoul, 05505 Republic of Korea

## Abstract

Genetically deregulated tumor cells generate vascular channels by vasculogenic mimicry (VM) that is independent of endothelial blood vessels. The morphological characteristics of VM and the role of EphA2 in the formation of VM were evaluated in 144 clinical samples of gastric adenocarcinoma and AGS gastric cancer cell line. It has long been believed that VM consists of PAS-positive basement membrane and CD31/CD34-negative cells. Interestingly, we found that the luminal surface of gastric tumor cells that form VM channels showed PAS-positive reaction, and that the involvement of CD31/CD34-positive tumor cells in the formation of VM channels. Highly aggressive tumor cells that formed VM were found to express CD31 or CD34, implicating the angiogenic and vasculogenic potential of the genetically deregulated tumor cells. VM occurrence was positively correlated with high expression of EphA2 in our patient cohort, and the indispensable role of EphA2 in VM formation was identified by gene silencing in AGS cells. We also report that Epstein–Barr virus (EBV)-positive tumor cells were involved in the formation of VM channels in EBV-associated gastric cancer samples. Overall, our results suggest that EphA2 signaling promotes tumor metastasis by inducing VM formation during gastric tumorigenesis.

## Introduction

Vasculogenic mimicry (VM), in which tumor cells create their own fluid-conducting channels without the involvement of endothelial cells^1^, was first described in human uveal melanomas as periodic acid-Schiff (PAS)-positive patterned vascular channel networks^2^. VM is considered as a plasticity of aggressive cancer cells in which *de novo* vascular networks form for the perfusion of rapidly growing tumors^3^, and is associated with malignant phenotype and poor clinical outcomes^4^. Although the initial description of VM was challenged^5^, it has been subsequently observed in several malignant tumors such as prostate cancer^6^, hepatocellular carcinoma^7^, breast cancer^8^ and gastric cancer^9^. Recently, a meta-analysis reported tumor VM to be associated with poor prognosis of gastric cancer^10^.

Initially, VM was believed to consist of PAS-positive extracellular matrix (ECM) on the inner wall of microcirculatory channels lined externally by tumor cells^11^. Later, a mosaic vessel model, forming the luminal surface of both tumor cells and endothelial cells, was introduced to describe the development of VM^12^. In an *in vitro* study using 3D-culture assay, mixture of gastric cancer cells and HUVEC cells formed mosaic vessels on Matrigel, implicating these vessels to serve as a bridge for the transfer of nutrition between endothelial cells and VM vessels^13^. In addition, accumulating evidence suggest close correlation between cancer stem cells (CSCs) and VM formation during carcinogenesis, and show that CSCs have the ability of trans-differentiation into vascular non-endothelial cells, thereby inducing VM^14,15^. The immunohistochemical expression of two endothelium-related proteins (CD31 and CD34) has been described in human aggressive malignant melanoma and their immunoreactivity could be related to the increased expression of genes involved in vasculogenic mimicry^16^. Despite the profound implication of tumor vascularization in tumor growth and metastatic dissemination^11,17^, little is known about the formation of VM in tissue samples from patients with gastric adenocarcinoma.

EphA2, an erythropoietin-producing hepatocellular (Eph) family member of receptor tyrosine kinases, has long been correlated with the growth of malignant tumors, including gastric cancer^18^. Over-expression of EphA2 and its ligand ephrinA1 has been shown to be an independent prognostic factor in post-operative gastric adenocarcinoma^19^. Moreover, a number of studies have demonstrated a pivotal role of EphA2 in the expression of VEGF protein^20^, and Epstein–Barr virus (EBV) infection as an EBV epithelial cell receptor^21,22^. Although EphA2 signaling is one of the key determinants in tumor microcirculation, its functional contribution to VM formation in gastric cancer remains unclear.

Therefore, our study aimed to examine the morphological characteristics of VM structure and tumor neo-vascularization in human gastric adenocarcinoma tissues, and to evaluate the correlation of EphA2 expression with VM formation, in order to explore the role of EphA2 signaling in the acquisition of VM structures in gastric cancer microenvironment.

## Results

### CD31-PAS reaction for vasculogenic mimicry structure

**Figure 1:**
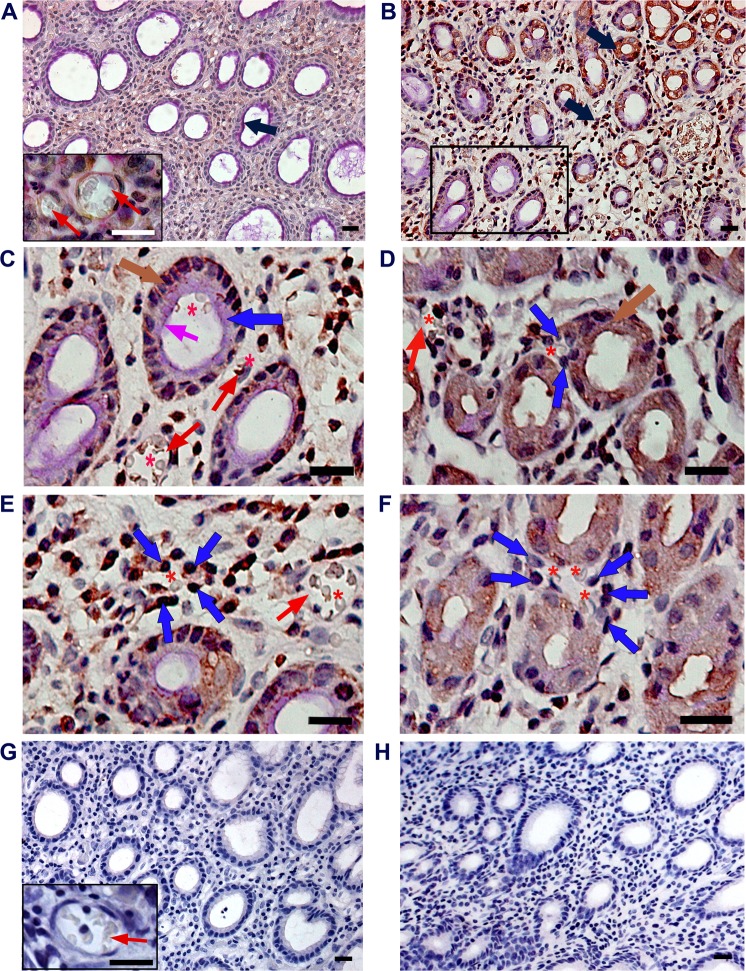
CD31-PAS double staining for vasculogenic mimicry. (**A**) Representative image of CD31-periodic acid-Schiff (PAS) reaction in tumor-adjacent normal gastric mucosa. The arrow indicates PAS reaction on the luminal surface. Red arrows in inlet show the blood vessels lined by CD31-positive endothelial cells. **(B)** CD31-positive glandular and stromal cells (arrows) in cancer tissue. **(C)** Large tubular-type vasculogenic mimicry (VM) channel (blue arrow); PAS reaction in the luminal surface (pink arrow); CD31-immunoreaction (brown arrow). **(D)** Tubular-type VM channel comprising of CD31-positive cuboidal tumor cells (blue arrows); CD31-immunoreaction (brown arrow). **(E**,**F)** Tubule-like VM channels; CD31-positive cuboidal tumor cells (blue arrows); blood vessels (red arrows). red blood cells (asterisks). **(G)** Negative control image of CD31/PAS double staining in tumor-adjacent normal gastric mucosa. Red arrow in inset shows the blood vessel lined by CD31/PAS-negative endothelial cells. **(H)** Negative control image for CD31/PAS in cancer tissue. All scale bars = 20 µm.

To examine the VM structure, CD31-PAS double staining was performed, as recommended by Maniotis *et al*. (1999), who had first described VM in melanoma. CD31-PAS reaction was investigated using sections of gastric cancer tissues, and adjacent non-neoplastic tissues (matched to normal gastric mucosa) from 144 patients with gastric adenocarcinomas. In normal gastric mucosa consisting of heterogenous mucosal cells, PAS reaction was observed in the cytoplasm and luminal surface of the gastric glandular epithelium (Fig. 1A, arrow). Typical blood vessels (Fig. 1A inset, red arrows) showed positive labeling of CD31 in their endothelial cells and PAS-positive reaction on the walls surrounding the endothelial cells. In gastric cancer tissues, surprisingly, the CD31-staining result was unexpected. In addition to vascular endothelial cells, CD31-positive reaction was observed on the glandular epithelium and some stromal cells (Fig. 1B, arrows). In Fig. 1C (the enlarged image of the rectangle in Fig. 1B), erythrocytes (asterisk) observed in the lumen of the large tubule (blue arrow) comprised of cancer glandular cells showing PAS reaction in the luminal surface (pink arrow) and CD31 immunostaining in the cytoplasm (brown arrow). The nearby blood vessels (Fig. 1C, red arrows) showed positive labeling for CD31 in their endothelial cells and PAS-reaction on their walls. Figure 1D shows a newly formed small tubular-type of VM channel (blue arrows) containing an erythrocyte (asterisk), as if the VM channel had sprouted from the CD31-positive cancer glandular cells (brown arrow). In another cancer stroma, tubule-like forms of VM were found. CD31-positive stromal tumor cells (Fig. 1E, blue arrows) encircled a red blood cell (Fig. 1E, asterisk). The three erythrocytes (Fig. 1F, asterisks) enclosed by PAS-positive cuboidal tumor cells (Fig. 1F, blue arrows) were found. We considered all these cases in which the erythrocytes were encircled by CD31-positive or PAS-positive cuboidal cancer cells, but not by attenuated endothelial cells, as VM-positive. To verify the specificity of the CD31 antibody and PAS reaction, negative controls were prepared in tumor-adjacent normal gastric mucosa (g) and cancer tissue (h). Negative reactions for CD31 and PAS on endothelial cells of the blood vessel (red arrow in inset) were identified.

### CD34-PAS reaction for vasculogenic mimicry structure

**Figure 2:**
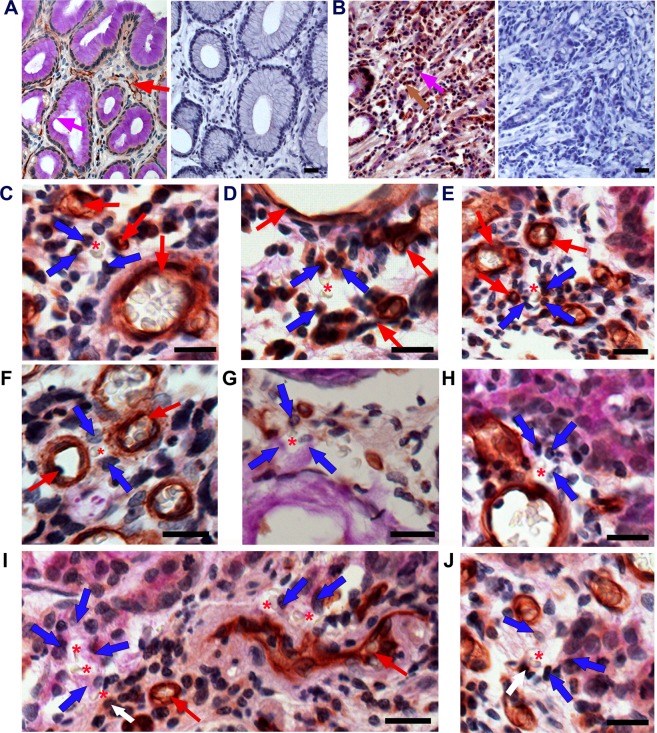
CD34-PAS double staining for vasculogenic mimicry. (**A)** periodic acid-Schiff (PAS) reaction (pink arrow) and CD34-positive blood vessels (red arrow) in normal gastric mucosa. **(B)** CD34- (brown arrow) and PAS-positive cells (pink arrow) in gastric cancer stroma. **(C**–**E)** Tubule-like VM channels; CD34-positive cuboidal tumor cells (blue arrows); blood vessels (red arrows); erythrocytes (asterisk). (**F**) Tubular-type VM; CD34-positive cuboidal tumor cells (blue arrows). (**G**,**H**) PAS-positive tumor cells (blue arrows). (**I**,**J**) PAS-positive tumor cells (blue arrows); CD34-positive small cuboidal cells (white arrow); erythrocytes (asterisks); blood vessels (red arrows). All bars = 20 µm.

The endothelial cell marker CD34, also known as hematopoietic progenitor cell antigen CD34^23^, was used for the identification of VM. CD34-PAS staining in normal gastric mucosa (Fig. 2A) showed strong reaction with PAS in the glandular epithelium (pink arrow) and with CD34 in the endothelial cells constituting typical blood vessels (red arrow). The negative control staining (right panel) did not show positive reaction for CD34 or PAS in normal gastric mucosa. In tumor stroma (Fig. 2B), CD34-positive mucosal cells (brown arrow) were found intermingled with PAS-positive cells (pink arrow). Right panel shows the representative image of negative control staining for CD34 and PAS reaction in cancer tissues. At high magnification, in the cancer stroma (Fig. 2C–E) near the typical blood vessels (red arrows), erythrocytes (asterisks) were seen surrounded by CD34-positive cuboidal tumor cells (blue arrows). Figure 2F shows a small tubular-type of VM channel containing a red blood cell (asterisk), consisting of two cuboidal cells expressing CD34-positivity (blue arrows) between typical blood vessels (red arrows) in the cancer stroma. We also detected tubule-like form of VM channel composed of PAS-positive large cuboidal tumor cells (blue arrows), enclosing erythrocytes (asterisks) in the cancer stroma, as exhibited in Fig. 2G,H. Images of Fig. 2I,J demonstrated many other forms of VM, consisting of PAS-positive large cuboidal cells (blue arrows) as well as CD34-positive small cuboidal cells (white arrow). We identified all these cases, with CD34-positive or PAS-positive vascular channels containing erythrocytes formed by gastric tumor cells, as VM-positive structures.

**Figure 3:**
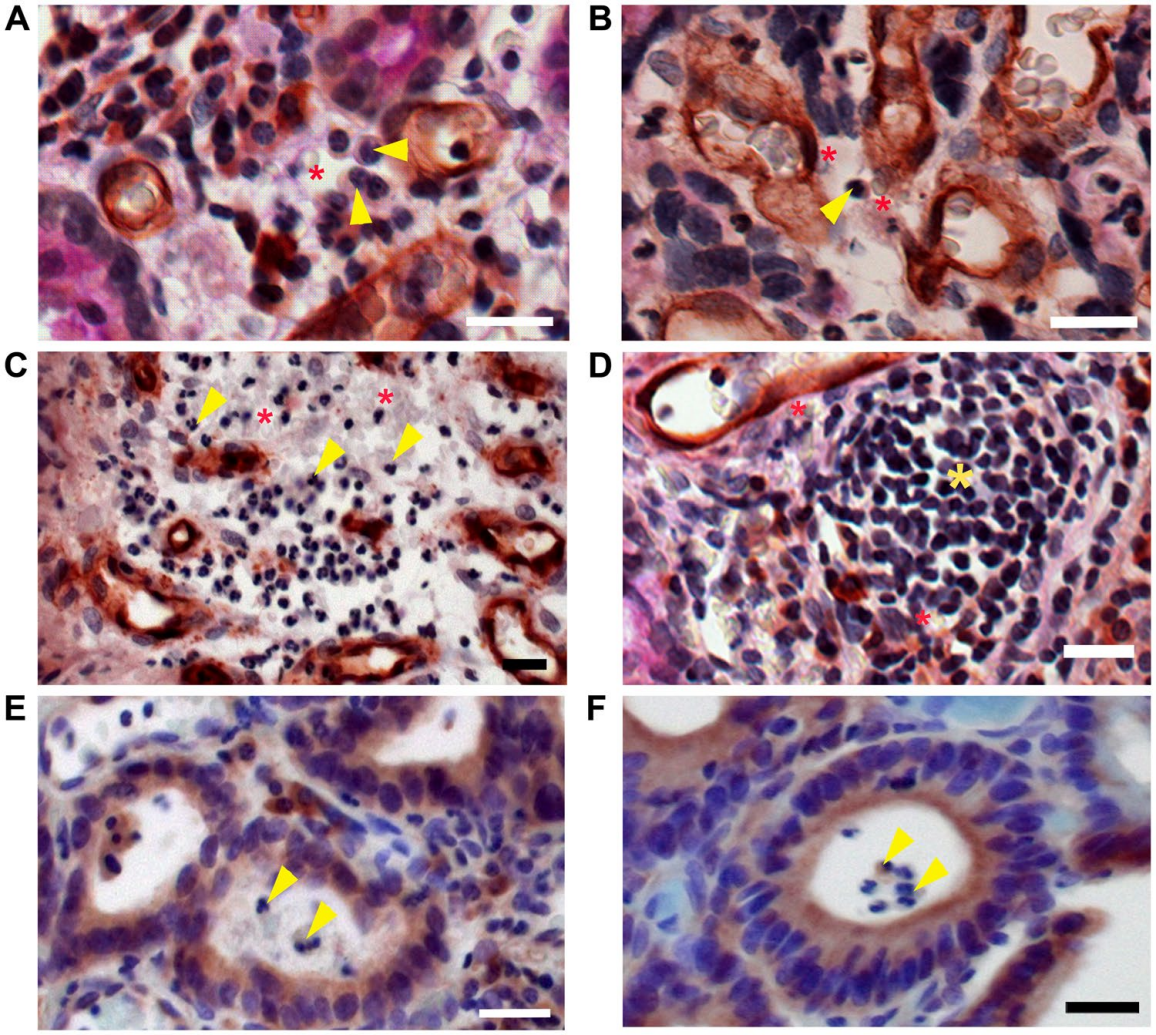
Inflammatory vascular leakage excluded from vasculogenic mimicry. (**A**–**D**) White blood cells (yellow arrowheads) or lymphatic nodules (yellow asterisk) near erythrocytes (red asterisks) in CD34-PAS-stained cancer tissues. **(E**,**F)** White blood cells (yellow arrowheads) in the glandular lumen in EphA2-immunostained cancer tissues. All bars = 20 µm.

However, if white blood cells were present with erythrocytes in the cancer stroma, it was regarded as extravasated blood in case of inflammation. As shown in Fig. 3A,B, lymphocytes (arrowheads) were observed near erythrocytes (asterisks). Occasionally, a large number of erythrocytes with white blood cells (Fig. 3C) or lymphatic follicles (yellow asterisk) near the erythrocytes (asterisks) were discovered (Fig. 3D). Large glandular lumens containing white blood cells (arrowheads) were detected in gastric cancer mucosa (Fig. 3E,F). All these cases were excluded from the consideration of VM-positive structures.

### Mosaic vessels in gastric cancer

**Figure 4:**
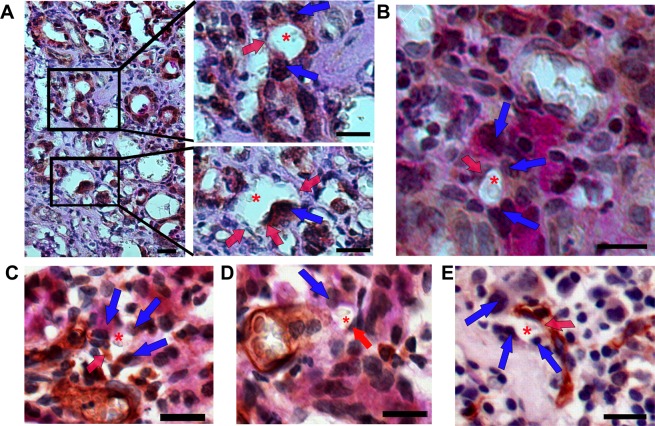
Mosaic vessels in gastric cancer tissues. (**A**,**B)** PAS-positive cuboidal tumor cells (blue arrows) and CD31-positive endothelial cells (red arrows) are situated in the vessel walls containing erythrocytes (asterisks). **(C**–**E)** PAS-positive tumor cells (blue arrows); CD34-positive endothelial cells (red arrows); erythrocytes (asterisks). All bars = 20 µm.

We also found mosaic vessels, in which some PAS-positive cuboidal tumor cells (blue arrows) and CD31-positive squamous endothelial cells (red arrow) were involved in the formation of erythrocyte-containing vascular channels (asterisk in Fig. 4A upper panel). In the lower panel, PAS-positive cuboidal tumor cells (blue arrow) were in the wall of the blood vessel lined by CD31-positive endothelial cells (red arrows). One small mosaic vessel, consisting of PAS-positive large cuboidal tumor cells (blue arrows) and CD31-positive endothelial cells (red arrow), was detected in the gastric cancer tissue (Fig. 4B). A similar observation was made in the tissues stained with CD34-PAS. As depicted in Fig. 4C–E, PAS-positive large cuboidal tumor cells (blue arrows) and CD34-positive endothelial cells (red arrow) enclosed a narrow lumen containing one erythrocyte (asterisk) in the tissues from patients with gastric adenocarcinoma.

### Association of EphA2 expression and vasculogenic mimicry

**Figure 5:**
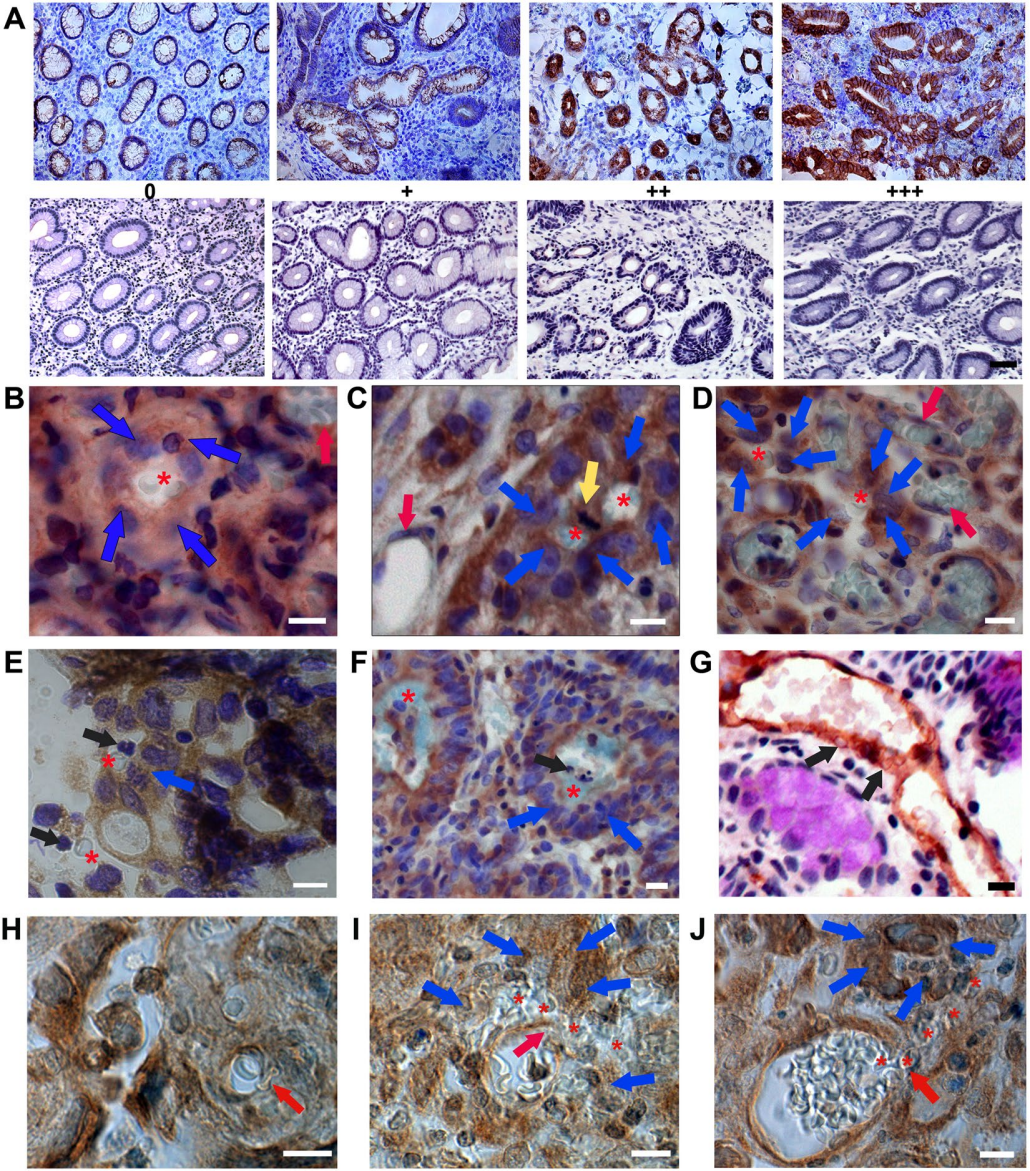
Immunohistochemistry of EphA2 in vasculogenic mimicry. (**A**) EphA2-immunohistochemical intensity score in the gastric adenocarcinoma. Lower panels show respective negative control images for EphA2 immunohistochemistry in matched cancer tissues. Bar = 50 µm. (**B**,**C**) Tubular-type of VM channels; EphA2-expressing cuboidal tumor cells (blue arrows); A tumor cell during mitotic division (yellow arrow). (**D**) Tubule-like VM channels; EphA2-expressing cuboidal tumor cells (blue arrows). Erythrocytes (asterisks); Blood vessels lined by endothelial cells, weakly expressing EphA2 (red arrows). (**E**,**F**) Inflammatory blood leakage; White blood cells (black arrows); EphA2-positive tumor cells (blue arrows); (**G**) Erythrocytes (black arrows) stuck to the vessel wall by CD34-PAS staining. (**H**) Erythrocytes (red arrow) leaking out of the cleft of vessel wall. (**I**,**J**) Leaked erythrocytes (asterisks) in the stroma. EphA2-expressing cuboidal tumor cells (blue arrows); Intercellular gap of the endothelial cells (red arrow). Bars in (**B**~**J**) = 10 µm.

To determine the relationship between EphA2 expression and VM formation, immunohistochemistry for EphA2 was conducted in the tissues derived from patients with gastric adenocarcinoma (n = 144). Staining intensity was graded as 0 (negative); 1 + (weak intensity); 2 + (moderate intensity); and 3 + (strong intensity) as shown in Fig. 5A. Negative control for EphA2 immunohistochemistry in matched cancer tissues are shown in the lower panels. The tubular-type of VM channels were also detected in EphA2 staining samples. In Fig. 5B,C, the tumor cells (blue arrows), external to the lumen containing erythrocytes (asterisk), showed strong intensity of EphA2. The yellow arrow in Fig. 5C indicates EphA2-positive tumor cells at the stage of cell division. Although weak EphA2 expression was detected in the endothelial cells (red arrows in Fig. 5B–D), we could identify VM channels, lined by large cuboidal tumor cells with strong EphA2 expression. Figure 5D demonstrated the tubule-like VM channels, containing erythrocytes (asterisk) enclosed by EphA2-expressing cuboidal tumor cells (blue arrows). However, when white blood cells (black arrows) were present in the lumen of VM channel formed by EphA2-positive tumor cells (Fig. 5E, blue arrow) or EphA2-positive glandular cells (Fig. 5F, blue arrows), along with erythrocytes (asterisks), the cases were excluded from VM count. In gastric cancer tissues, an interesting observation was that the erythrocytes stuck to the blood vessel wall (black arrows in Fig. 5G), as if to pass through the wall into the stroma. At high (1,000×) magnification, one erythrocyte (Fig. 5H, red arrow) was found to have leaked out of the capillary wall. We detected the leakage of erythrocytes (asterisks) through the intercellular gap of the endothelial cells (red arrow) and assumed the drained erythrocytes to enter the interstitial space, surrounded by the EphA2-expressing large cuboidal tumor cells (Fig. 5I,J, blue arrows).

To assess the clinical importance of EphA2 in the formation of VM, we investigated the correlation of EphA2 expression and VM incidence with clinicopathological features in 144 patients with gastric cancer. As shown in Table 1, among the 144 patients with gastric adenocarcinoma, 95 (66%) cases were identified as EphA2-high, and 49 (34%) were EphA2-low. EphA2 was highly expressed in advanced-stage disease with 32%, 19.4%, and 14.6% for III & IV stage, II stage, and I stage, respectively (P = 0.035). Statistically, high expression of EphA2 was found to be significantly correlated with tumor size (P = 0.0416), lymph node metastasis (P = 0.018), lymphovascular invasion (P = 0.013), and TNM stage (P = 0.035). Table 1 showed 38 tissue samples from 144 cases of gastric adenocarcinoma to be VM-positive (26.4%). As revealed by the χ2 test, VM-positive results were significantly correlated with tumor depth (P = 0.004), lymph node metastasis (P = 0.000), lymphovascular invasion (P = 0.004), perineural invasion (P = 0.003), TNM stage (P = 0.000), and cancer type (P = 0.001). In the VM-positive group (n = 38), the incidence of VM was 63.2% (24/38), 34.2% (13/38), and 2.6% (1/38) in III & IV stage, II stage, and I stage, respectively, demonstrating that significantly higher occurrence of VM was found in patients with high TNM stage.

**Table 1:** Correlations of EphA2 expression, VM and EBV infection and clinicopathological features in gastric cancer patients.

	N	EphA2		p value	VM		p value	EBV		p value
		Staining intensity								
		Low	High		Negative	Positive		Negative	Positive	
Age (year)				0.422			0.314			0.726
≤60	82	29 (20.1)	53 (36.8)		63 (43.8)	19 (13.2)		74 (51.4)	8 (5.6)	
>60	62	18 (12.5)	44 (30.6)		43 (29.9)	19 (3.2)		57 (39.6)	5 (3.5)	
Sex				0.802			0.894			0.040*
Male	96	32 (22.2)	64 (44.4)		71 (49.3)	25 (17.4)		84 (58.3)	12 (8.3)	
Female	48	15 (10.4)	33 (22.9)		35 (24.3)	13 (9.0)		47 (32.6)	1 (0.7)	
Tumor differentiation				0.275			0.583			0.754
WD + MD	39	10 (6.9)	29 (20.1)		30 (20.8)	9 (6.3)		35 (24.3)	4 (2.8)	
PD + Mucinous	105	37 (25.7)	68 (47.2)		76 (52.8)	29 (20.1)		96 (66.7)	9 (6.3)	
Tumor size				0.015*			0.135			0.278
≤5 cm	68	29 (20.1)	39 (27.1)		54 (37.5)	14 (9.7)		60 (41.7)	8 (5.6)	
>5 cm	76	18 (12.5)	58 (40.3)		52 (36.1)	24 (16.7)		71 (49.3)	5 (3.5)	
Depth of Tumor (T)				0.322			0.017*			0.565
T1	36	16 (11.1)	20 (13.9)		33 (22.9)	3 (2.1)		33 (22.9)	3 (2.1)	
T2	24	7 (4.9)	17 (11.8)		19 (13.2)	5 (3.5)		21 (14.6)	3 (2.1)	
T3	40	10 (6.9)	30 (20.8)		26 (18.1)	14 (9.7)		35 (24.3)	5 (3.5)	
T4	44	14 (9.7)	30 (20.8)		28 (19.4)	16 (11.1)		42 (29.2)	2 (1.4)	
Lymph node metastasis				0.020*			0.000*			0.004*
Yes	57	25 (17.4)	32 (22.2)		51 (35.4)	6 (4.2)		47 (32.6)	10 (6.9)	
No	87	22 (15.3)	65 (45.1)		55 (38.2)	32 (22.2)		84 (58.3)	3 (2.1)	
Lymphovascular invasion				0.038*			0.004*			0.114
Yes	85	22 (15.3)	63 (43.8)		55 (38.2)	30 (20.8)		80 (55.6)	5 (3.5)	
No	59	25 (17.4)	34 (23.6)		51 (35.4)	8 (5.6)		51 (35.4)	8 (5.6)	
Perineural invasion				0.174			0.011*			0.037*
Yes	73	20 (13.9)	53 (36.8)		47 (32.6)	26 (18.1)		70 (48.6)	3 (2.1)	
No	71	27 (13.9)	44 (30.6)		59 (41.0)	12 (8.3)		61 (42.4)	10 (6.9)	
TNM Stage				0.062			0.001*			0.248
IA + IB	42	20 (13.9)	22 (15.3)		40 (27.8)	2 (1.4)		36 (25.0)	6 (4.2)	
IIA + IIB	43	14 (9.7)	29 (20.1)		30 (20.8)	13 (9.0)		38 (26.4)	5 (3.5)	
IIIA + IIIB + IIIC	54	12 (8.3)	42 (29.2)		34 (23.6)	20 (13.9)		52 (36.1)	2 (1.4)	
IV	5	1 (0.7)	4 (2.8)		2 (1.4)	3 (2.1)		5 (3.5)	0 (0.0)	
Type				0.138			0.006*			0.914
EGC	35	15 (10.4)	20 (13.9)		32 (22.2)	3 (2.1)		32 (22.2)	3 (2.1)	
AGC	109	32 (22.2)	77 (53.5)		74 (51.4)	35 (24.3)		99 (68.8)	10 (6.9)	
Lauren’s classification				0.783			0.420			0.087
Diffuse	65	23 (16.0)	42 (29.2)		50 (34.7)	15 (10.4)		59 (41.0)	6 (4.2)	
Intestinal	48	14 (9.7)	34 (23.6)		36 (25.0)	12 (8.3)		41 (28.5)	7 (4.9)	
Mixed	31	10 (6.9)	21 (14.6)		20 (13.9)	11 (7.6)		31 (21.5)	0 (0.0)	

Abbreviations: WD, well differentiated; MD, moderately differentiated; PD, poorly differentiated; MU, mucinous adenocarcinoma; TNM stage, TNM Classification of Malignant Tumors. The stages were assigned in accordance with the American Joint Committee on Cancer-International Union for Cancer Control 7th edition. EGC, early gastric cancer; AGC, advanced gastric cancer. EBV, Epstein–Barr virus. Values are presented as number or percentage (%). *P < 0.05.

**Figure 6:**
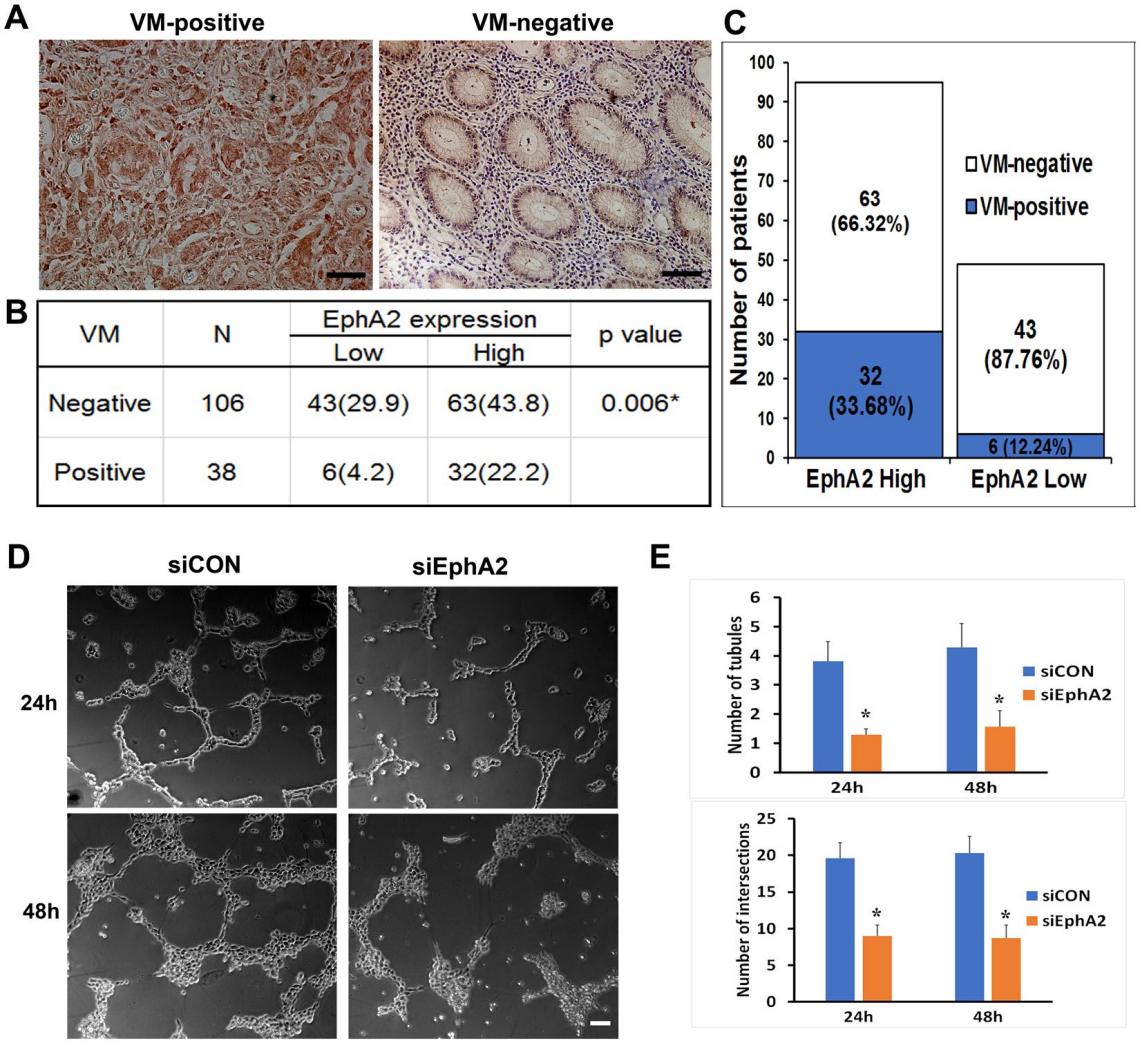
Correlation between EphA2 expression and vasculogenic mimicry. (**A**) Representative images of VM-positive tissue with high EphA2 expression, and of VM-negative tissue with low EphA2-intensity. Bars = 50 µm. **(B**,**C**) Table and graph showing the correlation between EphA2 expression and VM in patients with gastric adenocarcinoma (%). n = 144; *P < 0.05. **(D)** Representative images of VM formation in AGS cells transfected with non-silencing siRNA (siCON) or siRNA-EphA2 (siEphA2), grown for 24 and 48 h on the Matrigel with DMEM. Scale bar = 100 μm. **(E)** Graphs show the number of tubules and intersections in each experimental group. Values are mean ± SD from three independent experiments. *P < 0.01 vs. siCON.

In Fig. 6, the differential expression of EphA2 between VM-positive and VM-negative groups was evaluated. There was a significant correlation between high expression of EphA2 and VM-positivity (P = 0.006). In VM-positive group, 32 out of 38 cases (84.2%) showed high EphA2 expression (Fig. 6B). Figure 6C depicted that VM was present in 32 of the 95 EphA2-high samples (33.68%) and in 6 of the 49 EphA2-low samples (12.24%). These data suggest a positive association between EphA2 expression and VM formation in gastric adenocarcinoma specimens.

To determine the role of EphA2 expression in VM formation, VM tube formation ability of AGS gastric adenocarcinoma cells was investigated by silencing EphA2 gene using RNA interference technique. AGS cells transfected with siRNA EphA2 (siEphA2) or non-silencing siRNA (siCON) were cultured on Matrigel matrix with DMEM for 24 or 48 hours. When compared to siCON cells, siEphA2 cells showed reduced ability of tube formation in both group of 24 h and 48 h (Fig. 6D). EphA2-knockdown in AGS cells resulted in significant reduction of VM channel formation (Fig. 6E, P < 0.01 vs. siCON). These results suggest that EphA2 expression has a pivotal role in VM formation in AGS gastric cancer cells.

### Association of EBV-infection and vasculogenic mimicry

Recently, Epstein–Barr virus (EBV) infection has been shown to promote VM in nasopharyngeal carcinoma (NPC) and EBV-associated gastric cancer (EBVaGC)^24^. We determined the EBV status of our patient cohort by performing Epstein–Barr encoding small RNAs (EBERs) *in situ* hybridization in the tissue sections. As shown in Table 1, latent EBV infection was identified in 13 (9%) out of 144 patients, and EBV positivity was associated with male gender (P = 0.040), lymph node metastasis (P = 0.004) and absence of perineural invasion (P = 0.037).

**Figure 7:**
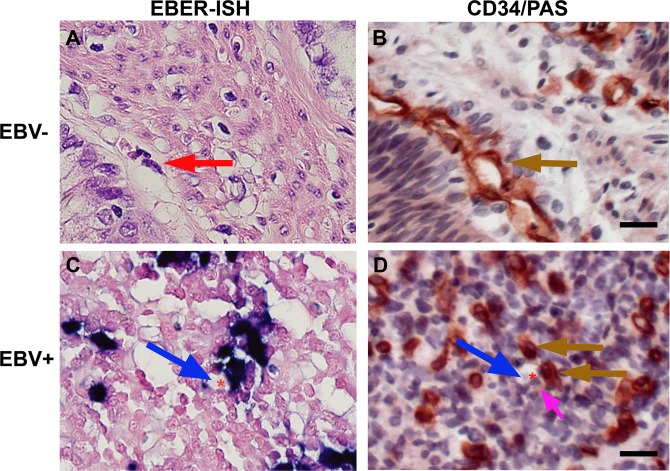
VM formation in the EBV-negative and EBV-positive gastric cancer sections. **(A)** EBV-negative gastric cancer tissues did not show any dark blue dots. (red arrow, blood vessel). **(B)** CD34 staining on the endothelial cells of blood vessels (brown arrow) in the section from the same EBV-negative patient in (**A**). **(C)** Dark blue dots in the nuclei of tumor cells showing EBV infection. VM channel (blue arrow) harboring EBER‐positive tumor cells was observed. (asterisk, red blood cell). **(D)** VM channel (blue arrow) harboring CD34-positive tumor cells (brown arrows) and PAS-positive cell (pink arrow) was observed in the section from the same EBV-positive patient in (**C**). (asterisk, red blood cell). EBV, Epstein-Barr virus; EBER, EBV-encoding RNA; ISH, *in situ* hybridization. Scale bars = 20 µm.

Next, we determined the correlation between EBV infection and VM formation by performing CD34/PAS double staining. The EBER-ISH image of Fig. 7A shows EBV-negative gastric cancer cells without any dark blue dots (EBV infection) in their nuclei. The endothelial cell-lined blood vessels (red arrow in Fig. 7A) showed CD34-positivity (brown arrow in Fig. 7B) in the section from the same EBV-negative gastric cancer patient. In Fig. 7C, multiple dark blue dots were observed in the nuclei of tumor cells, indicating that the specimen is EBV-associated gastric cancer (EBVaGC). EBV-positive dark purple cells were observed in the wall of VM channel (blue arrow) containing red blood cells (asterisk). CD34/PAS double staining in the section from the same EBV infected patient (Fig. 7D) revealed a VM channel (blue arrow) with red blood cell (asterisk) consisting of two cuboidal cells showing CD34-positivity (brown arrows) and PAS-positive cell (pink arrow). These data suggest that EBV-positive tumor cells may be involved in the formation of VM channels consisting of CD34-positive and PAS-positive tumor cells.

## Discussion

Ever since the first description of vascular mimicry (VM) in uveal melanoma^2^ as a novel paradigm for tumor perfusion, VM has been observed in various types of malignancies^6,7,14^ including gastric cancer^9^. In the previous studies, conducted for VM evaluation in gastric cancer specimens, VM was recognized as consisting of periodic acid-Schiff (PAS)-positive and CD31- or CD34-negative vessels, in contrast to classic endothelial blood vessels that are double positive for both PAS and CD31 or CD34^9,25,26^. PAS was shown to stain basement membranes^25,27^ and cytoplasm^9,26^ of gastric tumor cells lining the external wall of the VM channels without any involvement of CD31/CD34-positive vascular endothelial cells, like the golden standard for VM in solid tumors such as melanoma^2^, breast cancer^8^, and hepatocellular carcinoma^7^. In this study, PAS reaction was observed on the luminal surface and cytoplasm of the gastric tumor cells forming the VM channels. Two types of VM have been reported in tumors, i.e., a patterned matrix type and a tubular type^28^. The channels that are composed of the basement membrane of tumor cells are considered as patterned matrix type^2,7,9^. Meanwhile, tubular type-VM is identified in tumor cells lining the luminal surface of the channels^28^ and our results provide further supporting evidence (Figs 1C,D, 2F, 5B,C), suggesting that tubular type-VM occurs in gastric adenocarcinoma tissues.

The most obvious distinction between our results and those of previous studies is the involvement of CD31/CD34-positive cancer cells in the formation of VM channels. A growing body of research has indicated that CD31 or CD34 is not just used as endothelial marker. CD31, also known as platelet endothelial cell adhesion molecule 1(PECAM-1), is an integral membrane protein and a member of the immunoglobulin superfamily, which is also expressed on the surface of hematopoietic stem cells^29^ and undifferentiated embryonic stem cells^30^. We found a VM channel composed of CD31-positive tumor cells sprouting from pre-existing CD31-positive glandular epithelial cells in advanced gastric cancer tissue (Fig. 1D). Our finding was partly supported by a previous study that showed robust vasculogenic property of blood-derived CD31(+) cells^31^.

CD34 was first identified as a hematopoietic stem cell-surface antigen^23^, and an engraftment with highly purified CD34(+) cells had provided sufficient hematopoietic rescue for patients with myeloablation^32^. CD34 expression was found on the hematopoietic stem cells, mesenchymal stem cells, and endothelial progenitor cells, thereby suggesting that CD34 plays an important role in the differentiation of hematopoietic stem cells during both embryonic and adult hematopoiesis^33^. Based on the conserved expression of CD31 in the cells of hemangioblast lineage^29,31^, and considering the important role of CD34 in embryonic and adult hematopoiesis^23,32,33^, we hypothesized that CD31/CD34-positive cells have higher angiogenic and/or vasculogenic capabilities and might participate in the process of VM.

Many studies exploring the molecular mechanisms of VM indicated that VM formation is associated with cancer stem cells (CSCs)^34^, which have the capacity of trans-differentiation and acquisition of endothelial cell behavior^35^, as well as with vascular smooth muscle-like cells^28^. In addition, CSCs have the capability of trans-differentiation into vascular nonendothelial cells inducing VM^14,15^, suggesting a close relationship between CSCs and VM formation during carcinogenesis. CD34 has been detected on the tumor cells in glioblastoma, where VM phenomenon exists^36^. In melanoma study, CD31 and CD34 expression have been shown to be related to trans-differentiation of melanoma cells, thus implying that immunoreactivity for CD31 and CD34 is closely parallel with tumor progression and aggressive behavior of tumor cells^16^. These data might support our observation of higher expression of CD31 and CD34 in the glandular and stromal cells of tumor tissues compared to those in normal tissues. Our data showed the involvement of CD31- or CD34-positive cancer cells in the formation of VM channels and parallel expression of CD31 and CD34 with tumor progression, thereby suggesting that CD31 and CD34 enhance the channel-forming ability of gastric tumor cells. Therefore, CD31 and CD34 may be therapeutic targets for preventing VM-mediated gastric tumor growth and metastasis.

We found many cases of mosaic vessels, the walls formed by both large cuboidal tumor cells and squamous endothelial cells, nearby the traditional vascular vessels. Interestingly, red blood cells were seen to be leaking from the vascular vessels into the stroma surrounded by PAS-positive tumor cells. This finding might be suggestive of enhanced permeability of the host blood vessels in the tumor microenvironment, causing perfusion of the newly formed VM^37^ in gastric tumor stroma. VM is characterized by the ability of aggressive tumor cells to form vascular channels transporting fluid from leaky blood vessels or connecting with normal blood vessels^4^. Thus mosaic vessels indicate the connection between endothelial cells and tumor cells in blood vessel walls, and serve as a bridge to transfer nutrition and red blood cells between endothelial vessels and VM vessels^13^.

To understand the molecular mechanisms that regulate VM, the correlation between EphA2 expression and VM occurrence was investigated. The EphA2 receptor tyrosine kinase has long been associated with increased metastasis, poor prognosis, and decreased overall survival in malignant tumors including gastric cancer^18,19^. Our data with 144 patient tissues showed higher levels of EphA2 to be associated with advanced cancer stages, tumor size, lymph node metastasis, and lymphovascular invasion. Based on this finding, we suggested that EphA2 might influence the growth of malignant tumor by promoting metastatic motility of gastric cancer cells during gastric tumorigenesis. In this study, we showed that VM has a correlation with high grades of tumor malignancy and invasiveness, since increasing malignancy and higher aggressiveness need increased blood supply to compliment the rapid growth of the tumor^38^.

In addition, EphA2 was considered to be an epithelial cell receptor for Epstein–Barr virus (EBV) entry^21,22^, and EBV-infection has been shown to promote VM in nasopharyngeal carcinoma (NPC) and EBV-associated gastric cancer (EBVaGC)^24^. In EBVaGC samples, we observed that EBV-positive tumor cells were involved in the formation of VM channels harboring CD34-positive and PAS-positive tumor cells. We also found that VM was present in 6 (46%) out of the 13 EBVaGC samples and 32 (24.4%) out of the 131 EBV-negative gastric cancer samples. In order to obtain statistically significant results, however, further studies with a larger group of EBVaGC patients are needed to more extensively examine the correlation between EBV infection and VM occurrence.

Above all, VM in gastric adenocarcinoma is positively correlated with EphA2 expression, which is in accordance with the previous findings that EphA2 is involved in the formation of tubular networks by aggressive tumor cells in melanoma^39^, and that EphA2 is an important mediator of VM formation through the regulation of epithelial-mesenchymal transition^40^, EBV-infection^22^, EGF^21^, or EphA2/FAK/Paxillin pathway^41^.

In conclusion, our current study described the morphological details of VM in gastric adenocarcinoma, in which the heterogenous composition of mucosal cells complicate the identification of VM. Our results revealed that a tubular-type of VM, rather than a patterned matrix type, and the tubule-like VM, and mosaic vessels exist in advanced gastric tumor tissues. Interestingly, the aggressive tumor cells generating VM were found to express CD31 or CD34, probably because genetically deregulated tumor cells express angiogenic and vasculogenic markers. VM is positively correlated with high expression levels of EphA2, suggesting that EphA2 signaling may be involved in the promotion of tumor metastasis by VM formation during gastric tumorigenesis.

## Methods

### Patient samples

The study group comprised of a total of 153 patients, including 144 with gastric adenocarcinomas and 9 with gastrointestinal stromal tumor (GIST) as control, who underwent curative gastrectomy between May 2015 and April 2017 in the Department of Gastric Surgery, Asan Medical Center, University of Ulsan College of Medicine (Seoul, Korea). Of the 144 gastric cancer patients, 96 were males and 48 were females; their mean age was 58.45 years (range: 30–93 years). They were regularly followed for recurrent diseases, every 3 to 6 months after surgery. None of the patients received any anticancer therapy prior to sample collection. In addition, adjacent non-neoplastic tissues (matched to normal gastric mucosa), at approximately 5–20 cm from the tumor, were collected from each patient. All specimens were evaluated according to the tumor-node-metastasis (TNM) system recommended by the Union for International Cancer Control (UICC).

### Ethical permission

Written informed consent was obtained from each participant or participant’s legal guardian, and approval from the Institutional Review Board (IRB) of Asan Medical Center (2015-0370) was also obtained prior to this study. All procedures involving human participants were performed in accordance with the 1964 Helsinki declaration and its later amendments or comparable ethical standards.

### Immunohistochemistry for EphA2

Fresh specimens were immersed into 4% paraformaldehyde in phosphate buffer (77.4 ml of 1 M Na_2_HPO_4_ and 22.6 ml of 1 M Na_2_H_2_PO_4_ in 900 ml of distilled water) for 24 h and embedded in paraffin. The 5-μm paraffin sections were dewaxed and rehydrated. Antigen retrieval was achieved in Trypsin enzymatic antigen retrieval solution (ab970, Abcam, Cambridge, MA) at 37 °C for 20 min. After three washes with PBS, the endogenous peroxidase was blocked with 3% hydrogen peroxide, followed by incubation with 2% normal goat serum for 1 h to block any nonspecific binding. Anti-EphA2 antibody-rabbit monoclonal #6997 (Cell Signaling Technology, Danvers, MA), rabbit polyclonal ab5386 (Abcam, Cambridge, MA), SC-924 (Santa Cruz Biotechnology, TX), or mouse monoclonal SC-398832 (Santa Cruz Biotechnology, TX) was incubated with the sections overnight at 4 °C. After washing, the tissue sections were treated with biotinylated anti-rabbit secondary antibody, as appropriate, for 1 h, and then incubated with streptavidin-horseradish peroxidase (HRP) complex for 20 min. Finally, the sections were developed with diaminobenzidine (DAB) and counterstained with hematoxylin. For negative control, tissue sections were treated for overnight with pre-immune serum as a substitute for the primary antibody (EphA2) at the same concentration (1:1000)^42^. Subsequent procedures from secondary antibody treatment to counterstaining with hematoxylin were carried out according to the routine immunohistochemistry methods.

### Evaluation of EphA2 expression

EphA2 immunostaining was evaluated by two independent researchers who were blinded to clinical outcome. Adjacent normal gastric mucosae were used as controls. The proportion of stained cells was scored as: 0 (<5%), 1 (5% to 25%), 2 (26% to 50%), and 3 (>50%). Staining intensity was graded as 0 (negative), 1 + (weak intensity), 2 + (moderate intensity), and 3 + (strong intensity); followed by the summation of two scores. Final scores of <3 were classified as “EphA2-low” and those ≥ 3 as “EphA2-high”.

### CD31/34-periodic acid Schiff (PAS) double staining for VM

Sections were stained for CD31, using a polyclonal rabbit anti-human antibody (ab59251, Abcam, Cambridge, MA), and CD34, using a monoclonal mouse anti-human antibody (ab8536, Abcam, Cambridge, MA), to visualize blood endothelial cells. These were allowed to react with the appropriate secondary biotinylated-antibody for 1 h, followed by incubation with the HRP complex for 20 min. The sections were incubated with 0.5% PAS solution for 5 min, and further stained with Schiff reagent for 15 min, followed by rinsing in distilled water. For negative control, sections were treated with pre-immune serum as a substitute for the primary antibodies (CD31 or CD34) at the same concentration (1:1000). The routine immunohistochemical procedures were performed except for PAS reaction. Counterstaining with hematoxylin was completed before mounting. For each patient, more than three tissue sections were analyzed.

### EBER ***in situ*** hybridization

EBER-ISH was performed on the tissue sections using the BenchMark XT automated slide stainer (Ventana Medical Systems, Tucson, AZ, USA). The sections were treated with protease 2 (catalog no. 780-4147; Ventana Medical Systems) and labeled with an EBER probe (catalog no. 780-2842; Ventana Medical Systems) for 2 h. Hybridization products were visualized with Ventana ISH iView Blue Detection Kit (Ventana Medical Systems) according to the manufacturer’s instructions. Dark blue dots at the site of hybridization (nucleus) was regarded as positive for EBV.

### Vasculogenic mimicry tube formation assay

A 24-well culture plate was coated with 0.1 ml (50 μl/cm^2^) of growth factor-reduced Matrigel (Geltrex® LDEV-Free reduced growth factor basement membrane matrix), which was allowed to polymerize for 30 min at 37 °C. AGS gastric adenocarcinoma cells (1 × 10^5^ cells/well) were seeded on the solid gel and incubated with DMEM containing 10% fetal bovine serum for 24 or 48 hours. The number of tubules and intersections in 5–7 random fields were counted at 100X magnification in Image J 1.50i (NIH), and the results were reported as mean ± SD.

### EphA2 gene knockdown using siRNA

siRNA for EphA2 was obtained from Santa Cruz (sc-29304; Santa Cruz Biotechnology, CA, USA). The target sequence was 5′-AATGACATGCCGATCTACATG-3′ (EphA2)^43^, and non-silencing siRNA sequence 5′-AATTCTCCGAACGTGTCACGT-3′ was used as negative control. AGS cells (5 × 10^5^ cells/well) were transfected with siRNA at a final concentration of 20 to 100 nM using lipofectamine-2000 (Invitrogen). Following 8 h of transfection, cells were replaced with fresh medium containing 10% serum. Forty-eight hours after siRNA transfection, suppression of EphA2 expression was confirmed by western blot.

### Statistical analysis

All data in this study were evaluated using SPSS version 13.0 software (SPSS Inc.). The chi-square (χ2) test (for linear trends) and Fisher’s exact test were utilized to determine the relationships between EphA2 expression, VM, and clinicopathological features of the patients. Statistical significance was considered at p-values < 0.05. The *in vitro* data were analyzed using one-way analysis of variance with Bonferroni’s multiple comparison exact probability test. Statistical significance was set at p < 0.01.

### Ethical approval and informed consent

All procedures performed in studies involving human participants were approved by the Institutional Review Board (IRB) of Asan Medical Center (2015-0370) and in accordance with the ethical standards of the institutional and/or national research committee and with the 1964 Helsinki declaration and its later amendments or comparable ethical standards. Written informed consent was obtained from all participant or participant’s legal guardians.
